# “It felt very special, it felt customised to me”—A qualitative investigation of the experiences of participating in a clinical trial of CBT for young people at risk of bipolar disorder

**DOI:** 10.1111/papt.12313

**Published:** 2020-10-16

**Authors:** Wendy Theresa Jones, Sarah Peters, Rory Edward Byrne, David Shiers, Heather Law, Sophie Parker

**Affiliations:** ^1^ The Psychosis Research Unit Greater Manchester Mental Health NHS Foundation Trust UK; ^2^ Division of Psychology and Mental Health University of Manchester UK; ^3^ Primary Care and Health Sciences Keele University UK; ^4^ Youth Mental Health Research Unit Greater Manchester Mental Health NHS Foundation Trust UK

**Keywords:** bipolar, qualitative, bipolar at risk, randomized controlled trial, user‐led

## Abstract

**Objectives:**

The Bipolar at Risk Trial (BART) was a feasibility randomized controlled trial investigating cognitive behavioral therapy (CBT) compared with treatment as usual (TAU) in young people at high risk of developing bipolar disorder (BD). This qualitative study aimed to investigate participants’ experiences of trial involvement, and the acceptability of CBT for this population.

**Design:**

Participants were those identified as being at risk of bipolar disorder, determined by current symptoms or family history. A purposive sample of twenty‐one participants from both the intervention and TAU arms of the trial was recruited.

**Methods:**

Twenty‐one semi‐structured interviews were conducted by service user researchers (13 participants had received therapy and 8 TAU). Interviews were audio recorded with consent from participants and transcribed verbatim. NVivo 11 Pro software was used to conduct an inductive thematic analysis.

**Results:**

Super‐ordinate themes were “adaptability and flexibility,” “feeling understood and valued,” and “relevance of study and intervention” which had two sub‐themes—“value of the trial therapy” and “acceptability of trial processes.” Participating in the trial and having therapy enabled participants to feel understood and valued by research assistants (RAs) and therapists. Participants viewed therapy as relevant to their current concerns and valued adaptability and flexibility of RAs and therapists.

**Conclusions:**

Findings highlight the importance and value of flexibility, adaptability, and understanding in relationships between participants and trial staff. Findings also indicate that the trial processes and CBT focusing on mood swings are acceptable and relevant to participants from this at risk population.

**Practitioner points:**

- Young people at risk of bipolar disorder value a flexible approach to assessments and therapy, developing a rapport with research assistants and therapists and opening up to them when they feel comfortable to do so.
- CBT focusing on coping with mood swings was acceptable to the majority of participants who received it and it was perceived as helpful in ways that were personal to each participant.

## Background

Bipolar disorder (BD) is a severe and potentially disabling affective disorder characterized by fluctuations in mood from depression to mania. In a survey in 11 countries, overall lifetime prevalence was found to be 0.6% for bipolar type I disorder, 0.4% for bipolar type II disorder, 1.4% for subthreshold BD, and 2.4% for bipolar spectrum disorders (Merikangas et al., 2011). The cost of BD in the UK in 2007 was £5.2 billion and this is estimated to reach £8.2 billion per year by 2026 (McCrone, Dhanasiri, Patel, Knapp, & Lawton‐Smith, 2008). Misdiagnosis and a long duration of untreated illness is common (Drancourt et al., 2013). This can be prolonged if the onset of symptoms is in adolescence, resulting in poorer outcomes (Berk, 2007). Therefore, early detection, diagnosis, and treatment are imperative. Early intervention could reduce the chance of a full future episode of BD, and reduce the long‐term disability, distress, and associated costs of BD.

Early intervention in BD is based on approaches used for people with psychosis‐spectrum experiences such as the “close‐in strategy.” This strategy identifies people at risk of psychosis during the sub‐clinical period and delivers interventions aimed at reducing transition to psychosis (McGorry, Yung, & Phillips, 2003; Yung et al., 1998). Based on this strategy, Bechdolf et al. (2010) developed criteria identifying young people considered at risk of experiencing a full BD episode which encompass three identification categories: Group One—those with subthreshold mania; Group Two—those with depressed mood with cyclothymic features, and Group Three—those with depressed mood and a first degree relative with a diagnosis of BD. Preliminary studies have assessed the predictive validity of the bipolar at risk (BAR) criteria (Bechdolf et al., 2010 and Bechdolf et al. 2014) and research shows the criteria are reliable and valid (Bechdolf et al., 2014; Scott et al., 2017; Conus, Macneil, & McGorry, 2014; Malhi, Bargh, Coulston, Das, & Berk, 2014). However, minimal evidence exists about effective treatment options for those meeting BAR criteria and requires investigation given the unique opportunity to intervene. The Bipolar at Risk Trial (BART; ISRCTN10773067) was a feasibility randomized controlled trial testing the acceptability of CBT_BAR_, a 25 session CBT treatment focusing on appraisals of low and high moods for participants fitting the BAR criteria. CBT_BAR_ is developed from the Think Effectively About Mood Swings (TEAMS) CBT approach (Mansell et al., 2014), based on an Integrative Cognitive Model (ICM) (Mansell, Morrison, Reid, Lowens, & Tai, 2007). This ICM proposes that people hold extreme and contradictory appraisals about internal states leading to recurrent styles of thinking, behaving, and feeling that maintain and escalate mood episodes. The CBT_BAR_ approach uses an individualized formulation derived from the ICM to reduce experiences of mood swings by targeting key appraisal change, enabling people to have a better quality of life, independently of mood state. This was considered appropriate for young people at risk of BD given the evidence that these appraisals show links with prospective bipolar symptoms, are associated with a risk or cognitive vulnerability factor for the development of BD (Kelly, Dodd, & Mansell, 2017), poorer social and occupational functioning (Keller et al., 1992; Searson, Mansell, Lowens, & Tai, 2012) and distinguish from unipolar depression (Dodd, Mansell, Sadhnani, Morrison, & Tai, 2010).

Clinical trials of early interventions in BD are important for both the potential economic and societal benefits, and the benefits for individual service users and their family members if therapy is found to be safe and acceptable. It is important to assess participant experiences of trial involvement to explore value and meanings attributed to the trial. Qualitative exploration may also inform researchers of ways to enhance engagement within a larger definitive trial. This paper aims to address the primary question of “what are the experiences of young people at risk of BD within a feasibility trial of CBT_BAR_ versus treatment as usual (TAU),” and the secondary question of “what are the participant’s experiences of the CBT_BAR_ intervention within this trial.” While there is existing qualitative literature regarding various clinical trial experiences (Byrne & Morrison, 2014; Gee et al., 2018; Joyce, Tai, Gebbia, & Mansell, 2017; Notley et al., 2015), this paper intends to give a voice to young people meeting BAR criteria (Bechdolf et al., 2010 and Bechdolf et al. 2014) and is unique since service user researchers were involved in the research at every step.

## Method

### Participants

Twenty‐one participants from the Bipolar at Risk Trial (BART) took part in this qualitative study. Thirteen participants received CBT_BAR_ + TAU and eight were allocated to TAU alone (see Table 1 for participant characteristics). Eight participants in receipt of CBT_BAR_ were female and five male; five TAU participants were female and three male. The age range of interview participants was 17–26 years at the time of the interview (mean = 20.86). Eligibility was assessed in a face‐to‐face appointment with a research assistant (RA). Participants all met BAR criteria (Bechdolf et al., 2010) in at least one of the following three groups: Group One—people with subthreshold mania; Group Two—those with depressed mood with cyclothymic features; and Group Three, those with depressed mood and a first degree relative with a diagnosis of BD.

**Table 1:** BART qualitative sub‐study participant demographics

Pseudonym	Gender	Ethnicity	Trial arm	Age on date of interview
Lucy	F	White British	CBT_BAR_	19
Michael	M	White British	CBT_BAR_	19
Thomas	M	White Others	CBT_BAR_	23
Samantha	F	White British	CBT_BAR_	25
Caroline	F	White British	CBT_BAR_	26
Stanley	M	White British	CBT_BAR_	23
Matilda	F	White British	CBT_BAR_	19
Philip	M	White British	CBT_BAR_	17
Lucas	M	White British	CBT_BAR_	25
Harriet	F	White British	CBT_BAR_	25
Shelley	F	White Others	CBT_BAR_	19
Charlotte	F	White British	CBT_BAR_	21
Monique	F	Mixed Block Caribbean	CBT_BAR_	17
Average				20.86
Bob	M	White British	TAU	24
Emily	F	White Others	TAU	23
Anna	F	White British	TAU	22
Beth	F	White British	TAU	17
Evan	M	White British	TAU	20
Jane	F	White British	TAU	17
Andrew	M	White British	TAU	18
Jade	F	Mixed White/Black African	TAU	19
Average				20.00

13 participants in the qualitative sub‐study of the Bipolar at Risk trial (BART) had received cognitive behavioral therapy (CBT_BAR_), and 8 had received treatment as usual (TAU). Interviews were done with participants of both genders and a range of ethnicities and ages.

Participants for the qualitative study were recruited from consenting participants in the BART trial (*n* = 76) where they were randomized to TAU alone (*n* = 39), or CBT_BAR_ + TAU (*n* = 37). CBT_BAR_ comprised of up to 26 sessions plus optional booster sessions. Therapy aimed to help participants manage mood changes and improve quality of life. Participants in both arms of the trial completed an assessment battery at baseline, six and 12 months including the Beck Depression Inventory (BDI‐II) and the Global Assessment of Functioning (GAF). Assessments were conducted face‐to‐face with an RA at a location of the participant’s choice.

All participants in the qualitative study had prior experience of mental health treatment, for example, psychological therapies, counseling, and substance misuse treatment from a range of different services. Previous services included Child and Adolescent Mental Health Services (CAMHS) (*n *= 9); Community Mental Health Teams (CMHT) (*n *= 5); Primary Care Psychology (*n *= 3); Early Detection and Intervention Team (EDIT) (*n *= 3); inpatient (*n *= 3) and voluntary sector (*n *= 1); and substance misuse services (*n *= 1). Almost half of the sample (*n *= 9, 43%) had previously received pharmacotherapy (antidepressant medication).

### The research team

Two service user researchers with experience of utilizing mental health services and with qualifications in psychology led the data collection and analysis. Both researchers have experience of receiving CBT in differing contexts and one also experienced receiving care coordination from a mental health service. Other members of this research team include a clinical psychologist, a qualitative methodologist, and a carer who was also a General Practitioner (GP). Involving a carer in the team widened the perspective when interpreting and analyzing data and brought to the research a further depth of understanding into the lives of service users and their relationships with others. All team members were involved in the design of the qualitative study and development of study materials.

### Service user involvement

Employing service user researchers with personal experience of using mental health services to conduct interviews may enhance such evaluations by reducing perceived power imbalances between the researcher and participant, enabling participants to open up more honestly about their experience of the trial (Veseth, Binder, Borg, & Davidson, 2017). Talking to peers may help normalize mental health problems and reduce the feeling of stigma (Tanskanen et al., 2011). Involving service user researchers also makes the research more collaborative and offers a more varied perspective on the data (Sweeney, Greenwood, Williams, Wykes, & Rose, 2013).

### Procedure

The trial received research ethics approval from the UK NHS National Research Ethics Service North West, Greater Manchester East (REC reference number 15/NW/0336). Participant sampling was purposive to seek maximum variance in age, clinical presentation, referral pathway, and treatment allocation. Participants were recruited from both the treatment and control arms of the study. Participants were invited to take part in an optional qualitative study by a member of the trial team following six‐month assessments (allowing for completion of the typical active treatment window). Participants were informed their data would be anonymized, and they had the right to withdraw from the interview study. Those expressing interest were provided with participant information and contacted by a service user interviewer. For those who consented, a face‐to‐face interview was scheduled at a time and place of their choosing.

Interviewers followed a topic guide (see supplementary materials) and explored participant experiences of involvement in the BART trial including referral, randomization, study materials (trial leaflet and participant information sheet) and assessment procedures, and acceptability and understanding of the CBT_BAR_. The topic guide questioning on recruitment and research processes was broadly the same for those who received CBT_BAR_ + TAU and TAU alone; however, there was an extra section for discussion of CBT_BAR_ for those who received it. Key questions for those who received CBT_BAR_ were “how did you find CBT,” and “how suitable do you think this therapy was for your concerns?” The topic guide was flexible, enabling participants to discuss aspects of the trial and topics important to them. Participants were reimbursed £20 for their time. Interviews ranged from 13 to 60 minutes (mean = 36.30). Interviews were digitally audio recorded on an encrypted Dictaphone and transcribed verbatim. Any identifying information (names and places) was removed.

### Analysis

The data were analyzed using Thematic Analysis (Braun & Clarke, 2006). A critical realist stance was adopted to gain insight into participants’ own experiences. NVivo Pro (QSR International, 2015, version 11) was used to manage the data during analysis for firstly becoming familiar with the data by repeated reading and line by line coding by the first author. Coding was inductive, allowing exploration of themes without use of a pre‐conceived hypothesis. Coding of transcripts from both the CBT_BAR_ + TAU and TAU alone arms was done within the same NVivo file. Following organization of the coding, patterns and interesting themes were searched across the data set and reviewed. A thematic map was developed that captured the coding and enabled it to be visualized. Themes were further defined and named. The coding was done by a service user researcher, and the developing analysis was discussed regularly with the wider research team who were familiar with the data corpus to enable agreement on thematic names. Perspectives of research team members from different backgrounds were considered with the aim of avoiding bias which may have been present if the analysis had been conducted by service user researchers only or clinical psychologists only. For example, service user researchers may have implicit bias for or against CBT based on their previous experiences.

## RESULTS

Findings were organized into three themes—“relevance of study and intervention,” “feeling understood and valued,” and “adaptability and flexibility.” Each is described in turn, with illustrative quotes.

### Relevance of study and intervention

Participants reported that participation in the trial was relevant to them and made sense within the context of their prior experiences which included help‐seeking and accessing mental health services, having taken antidepressant medication, their work or education, and having family members with experience of BD. The two sub‐themes were “acceptability of trial processes,” and “value of the trial therapy,” each described below.

#### Acceptability of trial processes

Opinions were also sought about the acceptability of terms used in the trial. One participant said the “bipolar at risk” term encouraged him to take part as it sounded relevant to his experiences (Lucas, CBT_BAR_), and another participant said that it was a “relief” to hear it (Thomas, CBT_BAR_) as it enabled him to know what treatment plans could be put in place and what kind of support networks to access. This suggests participants were motivated by finding out more about the potential of a diagnosis of BD.

Lucas, CBT_BAR_ – “It was good because if I’d never have seen the bipolar bit I wouldn’t have come on it because it was my doctor, he said you’re showing signs of bipolar but it’s a little more complicated than that, you can’t just say that, you have to see someone about it. So then when I saw it I was like I might as well give it a shot, maybe these guys have the answer.”

Participants reported that the process of referral was straightforward and that the process of involvement in the trial was positive. None of the interviewed participants expressed a negative view of the overall trial.

Bob, TAU ‐ “I’ve enjoyed it…It’s something new.”

The process of randomization was described as acceptable and participants felt that the scientific need for this was adequately explained by the RAs at the time of recruitment. While being allocated to therapy was unanimously viewed as positive, receiving TAU was not necessarily disappointing and for one individual resulted in a sense of relief.

Andrew, TAU ‐ “Well it didn’t really bother me to be honest, just like it lifted a weight off my shoulders.”

Motivations to participate included wanting to become more independent and wanting to receive CBT and services quickly. This meant being allocated to TAU could be disappointing for some participants if it was perceived as delaying access to CBT.

Beth, TAU ‐ “A bit like upset in a way but then I wouldn’t, I know like eventually I’ll get therapy, just not soon.”

Several participants had altruistic motives such as wanting to help others and improve services in the future through taking part in a trial. This could mitigate disappointment at being allocated to TAU as participants perceived wider benefits to trial involvement.

Matilda, CBT_BAR_ – “It [randomisation] didn’t really bother me just because I knew that I would be, regardless, I’d be helping people even if I did not get the therapy.”

Being asked questions in assessments was acceptable by participants as RAs did not put pressure on them to answer questions, and explained why the questions were asked. Some participants had been asked similar questions by mental health services so found them repetitive, however, some participants reported finding answering questions enjoyable.

Lucy, CBT_BAR_ – “I’d only had one two months ago from my original [mental health service] meeting so it was a bit repetitive, I mean the questions were slightly different cos that was more tailored to mood so that was helpful and she was really nice all the way through it, kind of explaining why certain questions have to be asked.”

Jade, TAU – “I quite enjoyed it really… It was pleasant, it wasn’t unpleasant talking about things that are a bit dark or whatever, so I think it was probably the researchers making it a comfortable experience.”

There were mixed prior expectations of the trial. Following the trial, participants felt their experience had met or exceeded expectations. No participants said that their experience of being a participant in the trial had been worse than their expectations, suggesting the trial processes were broadly acceptable.

#### Value of the trial therapy

For the 13 participants allocated to CBT_BAR_ + TAU, receiving CBT as part of the trial was viewed as being valuable and providing participants with a positive and helpful experience. Some participants had been initially cautious about what to expect from receiving CBT_BAR_ in the trial, based on previous negative experiences with therapists or counselors from other services, but then found the CBT helpful.

Monique, CBT_BAR_ – “I didn’t know whether I wanted to do it or not because at the time it was like I’d tried CAMHS, I’d tried everything, I didn’t really find it that helpful, but er it’s actually been pretty helpful yeah.”

The experience of spending time with a therapist was particularly valued. Homework was viewed as an opportunity to extend this interaction outside the CBT_BAR_ sessions, and having copies of graphs, charts, and diagrams provided a tangible way of revisiting these conversations.

Caroline, CBT_BAR_ – “I preferred to do it [homework] because it just felt like if I was having therapy say once a week I wouldn’t have to wait the week I’d feel like I was kind of having ongoing therapy while I’m doing the homework.”

Participants valued that they had noted individual positive changes which they attributed to receiving CBT_BAR_ as part of the trial. Valued changes included getting out of the house more, resuming education, feeling less anxious, and having greater ability to manage mood swings. They perceived that CBT_BAR_ had a positive impact on their everyday lives.

Caroline, CBT_BAR_ ‐ "I’m out of the house most of the time now mostly, I never used to go out before hardly, I would cancel appointments, rearrange appointments, constantly try and avoid appointments.”

Matilda, CBT_BAR_ – “I don’t think that anyone I have seen in my life has ever helped me as much as just the sessions I had with her did, so it was beneficial and the time spent with her didn’t compare to anyone that I had seen like school counsellors and college counsellors and other therapists and stuff… I just like, I guess, to say thank you to everyone involved because it’s been probably one of the most biggest experiences in my life that’s impacted me in the best way possible, so I couldn’t thank you guys enough for giving me a future to get up and finish my education and I could get a job and I can kind of go out.”

Participants reported how they found that CBT brought up strong emotions and could be distressing; however, this was viewed as a valuable healing process.

Charlotte, CBT_BAR_ – “All through my CBT I was crying constantly… but it really like helped like I needed to do it… and it wasn’t like torture, it was like a healing kind of thing”.

### Feeling understood and valued

Participants reported they felt understood and valued by RAs, and those who received CBT_BAR_ described being understood and valued by their therapist. Firstly, experiences with RAs will be discussed, followed by experiences of CBT_BAR_ sessions.

#### Feeling understood and valued in assessments

Participants in both CBT_BAR_ + TAU and TAU alone arms of the trial described finding the process of completing psychological assessments with the researcher at the start of the trial and at follow‐up visits as valuable. Participants viewed the researchers they encountered to be skilled with a good understanding of their experiences, and who made them feel comfortable by being personable and providing them with space and time. This helped participants to feel able to be open, honest and able to disclose their personal experiences which were sometimes distressing.

Samantha, CBT_BAR_ ‐ “Even with [the RA], even though she was not a therapist I was feeling, I felt very comfortable with her so I could be open and honest about my whole circumstances.”

Anna, TAU – “everyone that I’ve spoken to from BART is just lovely, like me and [RA] would just switch on to something else like something personal to me and her that’s like linked in and just gave you that little bit of extra trust with someone where you could go yeah I’m gonna tell you everything I’m gonna hold nothing back and you can take it from there, so it’s kind of really good to do that because I did open up more and more.”

Participants reported that they felt confident the information they provided would be treated confidentially. This further helped foster a sense of trust which facilitated disclosure.

Anna, TAU – “Within five minutes I was relaxed and you know felt confident to tell her things, I didn’t feel I had to hold anything back because I knew everything was confidential so yeah.”

Some participants reported opening up was difficult at first but became easier over time and it was helpful to speak about psychological issues.

Evan, TAU – “At the start of the project speaking to them I was quiet and blunt with my answers but then when I got to the end of it I was good then I sort of trusted them a bit more and then I just was open with my answers. Because sometimes when people ask me about my past I like to keep it in the past but then I realised sometimes it’s better to speak about it."

Participants felt the assessment questions adequately covered their range of high and low moods and therefore felt that questions were relevant to their experiences and their difficulties were understood by the RA.

Jade, TAU – “It really went into detail about like questions about having bipolar because most of the… most times people ignore the mania and the highs and stuff like that they only ask the odd question but [RA] went into detail on that which I thought was really good because it’s often misunderstood as well but the questions were like well they were asking the right questions basically I think.”

One participant said the questions were upsetting because she had never spoken to anyone about the topics before, and two other participants found questions upsetting but felt a benefit from answering them, suggesting a sense of understanding the need to discuss mood.

Anna, TAU – “I think that yes there was questions that did make me upset but, in the end, I felt so much better for talking about it”.

Matilda, CBT_BAR_ – “It was quite eye‐opening in a way because I kind of realise that like a lot more about myself in a way, I started kind of unlocking doors I kept shut for quite a long time. So, it did cause me quite a bit of distress but I was, I kind of knew that it gets worse before it gets better anyway.”

#### Feeling understood and valued in CBT sessions

Participants who received CBT_BAR_ within the study also discussed the importance of feeling understood and valued by the therapist. Some participants had previous negative experiences with psychological therapists through other services who they reported had come across as patronizing, for example. Participants valued receiving CBT in a context where they felt equal partners in the therapeutic work they were undertaking and described how the therapist worked to make them feel comfortable and able to open up to discuss and work on problems.

Michael, CBT_BAR_ – “by the time I got to [BART therapist] I’d been through all these different people and I was like urgh, and she was great, I said, I said this to her, I said you’re the first person that I don’t feel patronised by, I don’t feel uncomfortable I just felt on a level with like let’s do some, some work now, yeah.”

Participants also described how the therapist supported them as they practiced new skills and techniques in a way that felt safe and enabled them to use the skills after the CBT_BAR_ had finished.

Matilda, CBT_BAR_ – “It’s like riding a bike, she was kind of like my safety pedals like the actual pedals you get and I guess they’re kind of coming off now and I’ve got to ride my bike on my own now. And but you have still kind of helped me, kind of become adjusted as much as I can, and kind of make as much progress to help me kind of continue to ride my bike all over the place without necessarily her supporting me like she was doing.”

### Adaptability and flexibility

Adaptability and flexibility were important factors that may have influenced retention to the trial. Firstly, adaptability and flexibility of RAs are discussed, followed by the same of therapists.

#### Adaptability and flexibility of RAs

Participants appreciated the flexibility of timing and location of the various appointments involved in the trial which included baseline and follow‐up assessments with RAs. Some preferred having assessment meetings at their own home as they had difficulty getting to services or found it difficult to be relaxed in a clinical setting.

Caroline, CBT_BAR_ – “She said we could either meet at the doctors where at mine or anywhere comfortable to me or at my home, at that point I wasn’t wanting to leave the house so we agreed that we would meet at home which was better for me as well so yeah then she came.”

The lengthy assessment meetings were made more acceptable by having the option of spacing it out over multiple sessions and to take breaks.

Evan, TAU – “My concentration isn’t that great so they let me go for breaks but yeah it was quite good.”

#### Adaptability and flexibility of therapists

Participants also appreciated the ability to rearrange CBT_BAR_ sessions and being able to contact the therapist via telephone outside of sessions if extra help was needed.

Lucy, CBT_BAR_ – “She was really nice, she was really understanding whenever I cancelled because I was ill or if I had coursework I had to catch up on, she was always there if I needed her out of times, outside of kind of session times.”

Participants also appreciated the clinical flexibility of therapists within sessions, not being pushed to talk about topics that they did not wish to talk about, and having the opportunity to pause or end sessions.

Matilda, CBT_BAR_ ‐ “With [BART therapist] with me she said like, if you’re not feeling up to having an appointment at any point, like you can just rearrange or cancel it. You don’t have to have the appointments if you’re not feeling up to it. But you can kind of, we can try and just rearrange it when’s obviously best for you and if at any point you don’t want to talk about something or if any point you want to kind of end the sessions or pause it for a second and stuff like that’s okay, and I think that’s what kind of made me, kind of helped me through it.”

Lucas, CBT_BAR_ – “It wasn’t like any free service I’ve got before, it wasn’t like going to the doctors and being referred, yeah it was a lot more understanding to my situation… It felt very special, it felt customised to me basically.”

Some participants experienced social anxiety and in some cases noted their therapist went out with them to address this, for example to a library.

Shelley, CBT_BAR_ – “It made sure I left the house because I wasn’t really going outside very much… once with [therapist] we went to the library in [city].”

Participants consistently described how they felt the CBT_BAR_ was tailored to them, with therapists working on individual problem lists and focusing on goal setting with participants, developing personalized aims and achievable goals to work toward to positively impact on their individual life.

Michael, CBT_BAR_ – “A very personal therapy that works around what I want by identifying my goals and then through becoming more aware of myself and then identifying what areas need work on and then in what ways I can work on them.”

## Discussion

This study aimed to explore the experiences of participants taking part in BART. Themes of “relevance of study and intervention,” “feeling understood and valued,” and “adaptability and flexibility” were identified. These themes are discussed with relevance to trial processes and CBT_BAR_ sessions, followed by comparison to existing literature, clinical implications, and limitations of the study.

### Trial processes

The importance of positive and meaningful engagement with research staff is highlighted by these themes. Adaptability and flexibility of staff and tailoring trial processes to BAR individuals were essential for the trial to be acceptable to participants who often lead busy lives. Adaptability and flexibility within the trial were demonstrated by the ability of participants to rearrange appointments with RAs, take breaks during assessments and skip assessment questions if they did not want to answer them. These factors have previously been reported to improve engagement in a trial of individual and family CBT for young people at risk of psychosis (Izon et al., 2020).

It was important that participants felt understood and valued by RAs working on the study. The process of opening up to RAs during assessments was viewed as a positive experience by most participants. The rapport developed with RAs also helped to elicit relevant information about the participant and retain participants at follow‐up.

It was important to participants that trial processes were relevant to their current lives and they saw value in taking part. Interview participants in the TAU arm found being in the trial valuable for reasons including having the opportunity to discuss experiences with an RA, along with the altruistic notion of helping others and potentially improving services in the future through taking part. This suggests that adequate information about the trial was provided at the outset (Lynöe, Sandlund, Dahlqvist, & Jacobsson, 1991). Interviews also suggested the trial processes of randomization, assessments, and the intervention was broadly acceptable.

### CBT_BAR_ sessions

Adaptability and flexibility were further demonstrated in the trial as participants who received CBT_BAR_ were able to easily rearrange sessions with therapists, have appointments in a location of their choice, and have appointments outside normal working hours. Flexibility has previously been reported as a strength of the TEAMS CBT approach, helping to develop a strong therapeutic alliance (Joyce et al., 2017). Within CBT_BAR_ sessions, creation of individual problem lists and individual goal setting ensured work done in sessions and homework was relevant to each participant. Doing homework tasks were useful in helping participants apply learning from CBT_BAR_ sessions to the outside world. It was not particularly seen as “hard work,” unlike in a study of CBT for young people who were at risk of psychosis (Byrne & Morrison, 2014).

Feeling understood and valued by the therapists was key to being able to open up and talk about psychological issues. Participants felt comfortable to disclose personal issues and express their emotions in therapy sessions, suggesting a positive therapeutic relationship. This therapeutic skill may have contributed to the high retention rates in the trial and high number of sessions attended. Some participants reported previously difficult experiences with therapists where a lack of feeling understood occurred and a power imbalance led to the feeling of being patronized. This was similar to the qualitative findings of Le Surf and Lynch (2007) who reported that young people seeking counseling wanted to avoid being patronized. With BART therapists young people felt more equal and understood, resulting in a better relationship that was more conducive to effective CBT. Examples of positive changes included greater ability to accept and manage mood swings, the ability to leave the house more, and being more careful with finances. Although CBT_BAR_ evoked difficult emotions at times, it was considered a helpful process, and it was important to participants that the intervention was relevant to their current concerns.

### Comparison with existing research

This is the first qualitative study involving clinical trial participants meeting BAR criteria. While it is therefore impossible to compare the findings to directly relevant studies, the findings can be compared to other qualitative studies from trials with participants from related clinical populations including those with BD or who are at risk of psychosis. A qualitative study investigating experiences of CBT for BD found that “changes resulting from therapy” was a prominent theme in addition to “useful elements of therapy,” including having a person‐centered and caring therapist (Joyce et al., 2017). These aspects were also important in the current study. A systematic review of 10 qualitative studies investigated experiences of psychological interventions for BD (Davenport, Hardy, Tai, & Mansell, 2019); however, the studies reviewed included only participants over the age of 18 with a diagnosis of BD whereas the current study involves participants at risk of BD aged 16 to 25. Despite these differences, findings were similar as positive changes following the interventions were reported, including control of moods, change of perspective, mood recognition and mood stability, and helpful aspects of therapy included therapist expertise, specific techniques, structure and having the opportunity to talk. These factors may be important to both people at risk of BD and those with a diagnosis of BD. A systematic review of psychological interventions for young people at risk of BD (Perich & Mitchell, 2019) highlighted that more qualitative research in this area is required. The current study goes some way to addressing this need.

Byrne and Morrison (2010) found helpful interactions with staff involving communication of psychological distress were crucial to the recovery of young people in an early detection of psychosis service. Byrne and Morrison (2014) similarly found the theme “opening up” with staff was important to young trial participants considered to be at risk of psychosis. These findings are similar to those of the current study where being able to open up with an understanding RA and develop a therapeutic relationship with a therapist was highly valued.

Davison and Scott (2017) interviewed young people at risk of BD who were children of adults with BD, finding that one important theme was “acceptable models of support.” They favored community‐based services such as peer support and reported that stigma was a barrier to accessing support. They conclude that young people should be asked for their thoughts about helpful interventions, and the current study has aimed to do this.

### Clinical implications

There are important clinical implications of the qualitative results (see Table 2). The results suggest the trial was valuable to this help‐seeking population, even for participants in the TAU alone arm as they felt listened to and had the opportunity to open up with RAs to discuss their experience of both low and high moods. Young people at risk of BD would likely appreciate greater availability of services which address mood swings. Participants who received CBT_BAR_ + TAU appreciated aspects of therapy such as feeling comfortable with the therapist and not feeling patronized. Clinicians should aim to be mindful of their interactions with clients to ensure they convey understanding and warmth. A good rapport between trial RAs and participants is also vital and RAs should inform participants early on about the flexibility within assessments enabling the participant to feel more empowered, enhancing their trust in the RA and therefore disclosure of experiences. This may also enhance retention rates. The flexibility of RAs and therapists to attend a location convenient for the participants was appreciated; however, this is not always practical for under‐resourced services supporting large caseloads. The research process differs from services since trials actively seek referrals and time can be taken to adapt to the schedule of each participant whereas services can be too busy to enable this approach.

**Table 2:** Implications for participants and for practice and a future definitive trial

Theme	Implications for participant	Implications and recommendations for practice and future definitive trial
Feeling understood and valued	Opening up more when feeling understood and valued by RA and therapist enables them to derive more benefit from therapy. Putting in effort to collaborate with the therapist and share information will also help participants derive more benefit from therapy. Reduces feelings of isolation.	Maintain confidentiality as much as possible. Ensure personal information is secure. Ensure therapists are well trained and skilled in developing a trusting therapeutic relationship. Ensure therapists are non‐judgemental and use a collaborative working style.
Adaptability and flexibility	Helpful for the participant to be in a flexible trial that adapts to their needs as they often lead busy lives. Feeling more comfortable in assessments and therapy sessions. Having a flexible location of therapy allows participants to try out getting out of the house more if this is a difficulty of theirs.	Use flexible style within sessions tailoring content to problem list. Ensure homework is relevant to problem list. All staff should be flexible in terms of location and time of appointments. RAs should allow breaks in assessment or spread out the assessment over multiple meetings. Questions in assessments should be tailored toward people experiencing mood swings. Adaptability and flexibility increase retention in trial.
Relevance of study and intervention	Achievement of relevant goals. Learning to manage mood swings. Learning early warning signs and triggers. Increased understanding of problems through formulation diagrams. Perception of positive changes through therapy. Answering questions in assessments that are relevant to experiences. Reduces feeling of stigma knowing that other similar people are in the trial. Helping others through participating in a trial gives a positive feeling.	Set homework that is appropriate for participant’s personal goals. Recruit participants through appropriate means for the target population. Ensure therapy sessions are tailored to mood swings and participant’s own experiences. Normalize the experience of BD symptoms in therapy sessions. Ensure questions asked in assessments are appropriate and relevant to being at risk of BD. Use clear formulation diagrams and allow participants to have a copy. All staff should seek regular supervision from clinical psychologists.

Implications are listed for participants and also for clinical practice and a future definitive trial of cognitive behavioral therapy (CBT_BAR_) for young people at risk of bipolar disorder.

### Study limitations

A limitation of the study was that the views reported are largely positive and this may reflect bias in recruitment as participants who agreed to have a qualitative interview may be more likely to have had a positive experience of taking part in the trial. It would be preferable to additionally interview those who had a wider variation of experiences of the trial such as those who attended a low number of CBT_BAR_ sessions or follow‐up assessments. While the majority of participants had positive views of the research process and CBT_BAR_, it is important to seek any negative or ambivalent views. Ensuring representation from participants who had not improved on scores of depression and functioning would gain a more representative sample and may improve the validity of the findings. Also, the sample was predominantly white British. Greater ethnic diversity would have been preferable; however, the sample reflects the demographic of service users in the area the trial took place. Furthermore, while checking of codes from a service user perspective was done within the research team, member checking of codes and themes by participants would also have been useful to gain a wider perspective of the data.

### Conclusions

Many aspects of the BART trial were valued by participants including the chance to open up to an understanding RA about distressing experiences, the perceived helpfulness of flexible, personally tailored CBT_BAR_ sessions, and development of a close therapeutic relationship. The CBT_BAR_ provided as part of the trial was an acceptable intervention for those meeting BAR criteria. The study suggests a larger trial of CBT focusing on mood swings would be acceptable and feasible, and expansion of services to early intervention in BD may be beneficial to young people at risk of BD. Additional trials are needed to understand which treatments or interventions would be beneficial to this group and further qualitative research is needed to understand the acceptability of these approaches.

## Funding statement

This paper presents independent research funded by the National Institute for Health Research (NIHR) under its Research for Patient Benefit (RfPB) Programme (Grant Reference Number PB‐PG‐1013‐32044). The views expressed are those of the author(s) and not necessarily those of the NIHR or the Department of Health and Social Care.

## Conflicts of interest

DS is expert advisor to the National Institute of Health and Care Excellence (NICE) center for guidelines and a member of the current NICE guideline development group for *Rehabilitation in adults with complex psychosis and related severe mental health conditions*; Board member of the National Collaborating Centre for Mental Health (NCCMH); views are personal and not those of NICE or NCCMH. No other authors declare any conflicts of interest.

## Author contributions

Wendy Theresa Jones, MSc (Conceptualization; Data curation; Formal analysis; Investigation; Methodology; Writing – original draft; Writing – review & editing) Sarah Peters (Conceptualization; Formal analysis; Funding acquisition; Methodology; Supervision; Writing – review & editing) Rory Edward Byrne (Conceptualization; Data curation; Investigation; Methodology; Writing – review & editing) David Shiers (Conceptualization; Formal analysis; Methodology; Writing – review & editing) Heather Law (Conceptualization; Funding acquisition; Methodology; Supervision; Writing – review & editing) Sophie Parker (Conceptualization; Data curation; Formal analysis; Funding acquisition; Investigation; Methodology; Project administration; Resources; Supervision; Validation; Writing – review & editing

## Supporting information

Supplementary Material

## Data Availability

The data that support the findings of this study are available on request from the corresponding author. The data are not publicly available due to privacy or ethical restrictions.
