# Supplementary material for: “It felt very special, it felt customised to me”—A qualitative investigation of the experiences of participating in a clinical trial of CBT for young people at risk of bipolar disorder
Source: Psychol Psychother. 2020 Oct 16;94(3):686–703. doi: 10.1111/papt.12313 (PMC8451762; doi:10.1111/papt.12313)
Supplement: Supplementary file 1 — Supplementary Material [file PAPT-94-686-s001.docx]

**BART Trial: Qualitative study Topic Guide 1 (for participants)**

**Recruitment to the study**

- Can you tell me how you came to be involved in the study? *[How did you find out about the study? - probe for referral, expectations of the study, previous experience of research/therapy]*
- How did you decide whether or not to take part in the study? Was there anything you weren’t too sure about? Why do you think some people might decide not to take part? *[probe for extent to which they discussed decision with family/others]*
- What happened once you were referred? Who contacted you and what happened? *[probe for views of RA, study information/informed consent, screening and assessment procedures, randomisation]*
- Did you have any thoughts, positive or negative, about any terms used for this study – for example ‘Bipolar’, ‘At risk’? *[Any experience of these previously?]*

**Research process (Before/During)**

- The BART study is a randomised controlled trial. What do you understand by that term? What do you think the point of randomising is? What did you think when you were allocated [CBT/TAU]?
  - **Flexibility with other treatment/therapy?**
- When the RA goes through the questions she needs to ask you, and you complete questionnaires - How did you find that? Where there any questions that surprised you?
- Is there anything you weren’t asked about that you would have liked to be?
- Were there any changes in what you expected or hoped to get help with during the trial?

- *[prompt re 3/9-month follow-up call]*

**Therapy [for those allocated to CBT only]**

- How would you describe the therapy to someone else, like a family member?
- How did you find therapy? *[probe for views of therapist, content of sessions, length and timing of sessions, homework, materials, location]*
  - **Mechanisms of change?**
- How does it compare with any other treatments you’ve had for emotional or mental health concerns?
- How suitable do you think this therapy was for your concerns? What would you have liked it to be like?
- How was it when therapy came to an end? [if it has, or ‘How do you feel about therapy finishing?]
- How has your health been since the study? If anything’s changed (better/worse), what might be the reason for this? *[probe for any life-events, views of mechanism of action]*

**Research process (After)**

- Some people decided not to take part in, or finish the study. We are interested in understanding the reasons for this and how we could do things differently in future studies. What do you think might make some people reluctant to take part/drop out?
- [For non-completers] What factors influenced your decision to leave the study? Was there anything the research team could have done differently?]
- Is there anything else you’d like to discuss about taking part in the BART project?
